# Forecasting colorectal cancer screening in Japan: regional and sex disparities threaten national targets

**DOI:** 10.1007/s10147-026-03150-8

**Published:** 2026-07-27

**Authors:** Hasan Jamil, Aminu Kende Abubakar, Takao Suzuki, Tshewang Gyeltshen, Hellen Wairimu Babu, Phuong The Nguyen

**Affiliations:** 1 Hitotsubashi Institute for Advanced Study, Hitotsubashi University, 2-1 Naka, Kunitachi, Tokyo 186-8601 Japan; 2 Graduate School of Public Health, St. Luke’s International University, Tokyo, Japan; 3 Division of Population Data Science, National Cancer Center Institute for Cancer Control, Tokyo, Japan; 4 Global Health Policy Department, Graduate School of Medicine, School of International Health, University of Tokyo, Tokyo, Japan

**Keywords:** Colorectal cancer screening, Bayesian forecasting, Regional disparities, Sex disparities, Japan, Health Japan 21

## Abstract

**Objective:**

To forecast prefecture-level progress toward Japan's 60% colorectal cancer (CRC) screening target by 2028 and quantify regional and sex-based disparities.

**Methods:**

We extracted CRC screening rates (ages 40–69) for all 47 prefectures from 2013 to 2022 using the Comprehensive Survey of Living Conditions. Sex-stratified Bayesian linear regression models were applied to logit-transformed rates. Prefectures were considered likely to achieve the target if the probability of ≥ 60% uptake by 2028 was ≥ 80%.

**Results:**

Nationwide coverage is projected to reach 51.9% (95% CrI 44.9–58.8%) by 2028, falling 8.1 percentage points short of the 60% target. Forty of 47 prefectures (85%) had less than a 20% posterior probability of meeting the target, and 14 were projected below 50% coverage. Only Yamagata exceeded the 80% probability threshold for the total population (probability 0.988). For men, three prefectures did (Yamagata, Yamanashi, Miyagi), versus one for women. Men outperformed women in 41 of 47 prefectures, with a median sex gap in target probability of 8.0 percentage points and a median lag of 6 years in projected achievement.

**Conclusions:**

Based on 2013–2022 trends, Japan is projected to miss the 60% CRC screening target by over a decade. Most prefectures had less than a 5% estimated probability of reaching 60% by 2028, and women lag men by a median of 6 years. Incremental progress under the current system appears insufficient. Reaching the target will require accelerated, prefecture-specific, and sex-sensitive interventions beyond what the current system delivers.

**Supplementary Information:**

The online version contains supplementary material available at https://doi.org/10.1007/s10147-026-03150-8.

## Introduction

Colorectal cancer (CRC) is Japan's most frequently diagnosed malignancy and the second leading cause of cancer death, with over 150,000 new cases and 53,000 deaths annually [1]. Many of these deaths can be prevented through early detection via screening. Patients diagnosed at advanced stages have a 5-year survival rate below 20%, whereas survival exceeds 90% when CRC is identified early [2]. In our previous work, we projected that in Japan, CRC, together with lung cancer, will remain among the leading burdens of incidence and mortality through 2054, and CRC will also be one of the most prevalent cancers by 2050 [3, 4]. These projections highlight the urgent need to strengthen and expand access to screening to reduce CRC mortality.

Japan's national CRC screening program offers annual immunochemical fecal occult blood testing (FIT), recommended for all individuals aged 40 years and older [5]. Screening is delivered through three channels: municipality-based organized programs, employer-provided workplace screening, and voluntary health check-ups [5]. Organized population-based CRC screening in Japan is primarily FIT-based, with colonoscopy reserved as follow-up for positive results [6]. In voluntary health check-ups (*ningen dock*), colonoscopy may be offered as a primary screening test [6]. The CSLC measure likely captures a mixture of these modalities. Despite the availability of these options, uptake remains low [7]. The 2022 national survey reported coverage of only 49.1% among men and 42.8% among women. Substantial regional variation exists: Yamagata Prefecture reported the highest uptake (~ 65%), whereas Hokkaido (~ 38%) and Okinawa (~ 38%) had the lowest. Even Tokyo, the nation's capital, showed suboptimal participation (~ 50%) [8]. These disparities may have contributed to mortality differences between prefectures, with a greater than 3-year gap in life expectancy and healthy life expectancy between the highest- and lowest-performing regions, partly due to uneven reductions in cancer and other non-communicable disease (NCD)-related mortality [9]. Health improvements have also slowed more for women than for men [9]. Recent cohort evidence also shows earlier smoking initiation and persistently higher smoking prevalence in men than women in Japan, reinforcing sex-linked prevention gaps relevant to CRC [10]. Addressing these geographic and sex-based inequities is central to Japan's current cancer control strategy.

In response to rising chronic disease burdens, including cancer, the Japanese government introduced the National Health Promotion Movement in the 21st Century (Health Japan 21) in 2000 [11]. The first term established a foundation for population-level health promotion, and the second term (2007–2023) broadened targets to extend healthy life expectancy and reduce inequalities. The third term (2024–2035) aims to build a 'sustainable society where no one is left behind,' emphasizing inclusion, regional equity, and stronger program implementation [12]. It seeks to increase cancer screening participation by encouraging regular checks throughout adulthood and supporting local governments in improving delivery. Within this framework, Japan has set a target of at least 60% participation in all cancer screening programs by 2028 [12]. This benchmark was set as a policy target in the third-term *Health Japan 21* plan. This target builds on the earlier national benchmark of 50%, set in 2007 under the first Basic Plan to Promote Cancer Control Programs [13]. It applies to all five cancer screening programs (gastric, lung, colorectal, breast, and cervical) delivered by municipalities across Japan [14].

Achieving the third-term Health Japan 21 target requires understanding prefectural and sex-specific screening trajectories. Empirical evidence across Japan's 47 prefectures is limited. We recently analyzed Health Japan 21 tobacco targets and found pronounced regional disparities in progress, highlighting the same implementation challenges that can impede equitable gains in cancer screening [15]. Forecasting CRC screening uptake and the probability of meeting the target is critical for evaluating policy feasibility and guiding strategies to address regional and population-level gaps. This study aimed to estimate the probability of achieving the 60% national target at both national and subnational levels and quantify regional and sex-based disparities in uptake and projected progress.

## Methods

### Study design

We performed a retrospective ecological time-series study of colorectal cancer screening coverage in all 47 Japanese prefectures. For each prefecture we modelled coverage from the four triennial survey waves (2013, 2016, 2019, 2022). Analyses were stratified by sex (total population, men, women).

### Data source and participants

Self-reported prefecture-level screening coverage for adults aged 40–69 years was obtained from the prefecture level aggregated reports of Comprehensive Survey of Living Conditions (CSLC**)** screening uptake for the survey years 2013, 2016, 2019, and 2022 [16]. The 2007 and 2010 waves were excluded because the 2012 Cancer Control Plan revision and concurrent CSLC questionnaire changes produced a discontinuity in observed trends. The CSLC does not distinguish between organised screening (primarily FIT), workplace-based screening, opportunistic examinations, or diagnostic procedures prompted by symptoms, so reported coverage is a composite of all channels.

### Variables and outcomes

The primary outcome was colorectal cancer screening coverage, defined as the self-reported proportion of adults aged 40–69 years who underwent a colorectal cancer screening examination through any delivery channel (organised municipal, workplace-based, or opportunistic) within the year preceding each survey. The sole predictor in each prefecture-specific model was calendar year, centred at the first observed year to improve numerical stability; no additional covariates were available. Secondary measures included the probability that coverage would reach the national target of 60% by 2028, the annual percentage-point change (APC) in coverage derived from model slopes, and the calendar year in which the posterior probability of exceeding 60% first reached 80% (the target year).

### Statistical analysis

We applied a Bayesian linear trend to the logit of coverage for each prefecture and sex. Year was the sole predictor, centred at 2013. We placed weakly informative normal priors (mean 0, precision 0.001) on the intercept and slope, and a gamma(0.001, 0.001) prior on residual precision. The Bayesian approach produces direct posterior probabilities of target achievement, which is our primary outcome. The logit link constrains projections to valid proportions and is approximately linear over the observed coverage range (30–65%). We adopted a Bayesian modeling framework to estimate screening trends and generate projections. This approach allows uncertainty in model parameters to be fully propagated into projected estimates and enables direct estimation of the probability of achieving policy targets, which is particularly informative for public health planning [17]. Screening coverage was modeled on the logit scale to ensure that predicted values remained within the valid range (0–100%) and to stabilize variance across different levels of coverage [18]. Posterior samples were obtained using Markov chain Monte Carlo with three parallel chains, an initial warm-up of 2,000 iterations, and a total of 10,000 retained iterations. Convergence was assessed by visual inspection of trace plots. Posterior draws were converted back to percentages to report fitted values and projections.

Projections extended through 2028. For each prefecture and sex, we calculated the posterior probability that coverage in 2028 would be at least 60 percent. We classified a prefecture as likely to meet the national target when this probability was at least 80 percent. To summarize the pace of change, we reported the annual absolute change in coverage as percentage points per year, derived from model-based predictions on the probability scale.

### Validation

Models trained on 2013–2019 data generated out-of-sample predictions for 2022. Predictive performance was summarised by mean absolute error (MAE), bias (mean error), root mean square error (RMSE), mean absolute percentage error (MAPE), and the proportion of observations falling within 95% credible intervals. All inferences are expressed as posterior medians with 95% credible intervals; two-sided decision rules based on 95% intervals were adopted. All analyses used R (version 4.5.2) with JAGS (version 4.3.2). Analysis code is available at (https://github.com/hjamil-md/crc-screening-projection-japan).

## Results

In 2013, nationwide CRC screening coverage was 37.9% (men 41.4%, women 34.5%) (Table 1). Coverage was highest across the northeastern Tohoku region, led by Yamagata (54.9%), Miyagi (47.6%), and Akita (45.5%), and lowest in western Japan and the urban centres of Osaka (29.8%) and Fukuoka (32.1%), as well as Hokkaido (32.7%) in the far north. Men screened more often than women in every prefecture, with a mean gap of 6 percentage points.

**Table 1:** Colorectal cancer screening coverage in 2013 and 2022. *Source*: Comprehensive Survey of Living Conditions, e-Stat Japan

Prefecture	2013 (%)			2022 (%)			2028 projected, % (95% CrI)		
	Total	Men	Women	Total	Men	Women	Total	Men	Women
Nationwide	37.9	41.4	34.5	45.9	49.1	42.8	51.9 (44.9–58.8)	54.9 (47.1–62.6)	49.1 (41.3–57.1)
Hokkaido	32.7	37.9	28.2	38.1	44.0	32.8	42.4 (31.5–53.5)	48.7 (38.1–59.6)	36.9 (26.7–47.6)
Aomori	38.8	42.0	36.5	51.1	53.5	48.7	61.3 (42.5–77.4)	63.0 (42.8–80.3)	58.5 (45.1–70.9)
Iwate	43.9	44.0	42.7	52.9	55.0	50.6	58.2 (47.3–68.7)	62.6 (46.5–76.4)	54.3 (39.5–69.2)
Miyagi	47.6	51.5	44.0	55.2	57.5	52.7	60.4 (51.7–68.7)	61.4 (55.7–67.0)	59.2 (46.6–71.4)
Akita	45.5	48.4	43.6	50.3	51.8	47.8	53.5 (46.8–60.3)	54.5 (47.0–62.1)	50.6 (44.5–56.5)
Yamagata	54.9	57.1	52.8	64.7	65.1	63.1	70.5 (63.7–76.7)	69.8 (61.0–77.6)	68.8 (59.4–77.5)
Fukushima	43.9	46.6	41.0	50.5	53.1	47.4	54.8 (48.8–60.7)	57.3 (50.3–64.0)	51.8 (46.2–57.6)
Ibaraki	36.8	40.7	33.3	45.1	49.2	41.7	51.7 (38.9–65.0)	54.6 (46.7–62.0)	49.4 (31.2–68.8)
Tochigi	41.6	45.3	37.8	45.7	49.1	42.3	50.2 (38.0–63.3)	52.6 (42.2–62.1)	47.6 (30.1–66.2)
Gunma	38.5	42.6	34.6	46.3	49.1	43.5	52.9 (41.5–63.8)	55.0 (41.5–67.6)	51.0 (38.7–62.7)
Saitama	37.9	40.6	35.2	43.0	44.8	41.3	48.1 (35.2–61.1)	50.0 (33.5–66.4)	46.4 (36.7–56.1)
Chiba	40.0	43.6	36.7	46.2	48.5	44.2	50.7 (40.0–61.5)	52.1 (44.9–59.1)	49.7 (34.8–65.2)
Tokyo	39.9	43.2	36.6	49.8	51.7	48.0	57.9 (46.2–69.1)	58.9 (46.2–70.6)	57.1 (45.8–67.9)
Kanagawa	38.5	43.7	33.4	47.3	50.6	44.2	52.7 (45.0–60.1)	54.9 (48.0–62.1)	51.0 (42.8–59.3)
Niigata	45.0	47.9	42.2	53.6	56.2	50.9	59.7 (49.5–70.0)	62.8 (48.3–75.3)	56.3 (46.4–65.5)
Toyama	43.3	46.6	39.9	52.1	54.6	50.5	57.6 (51.1–64.1)	59.2 (48.1–69.7)	56.4 (46.0–66.8)
Ishikawa	39.0	40.8	37.5	45.5	50.0	42.7	51.8 (38.6–65.3)	58.4 (41.4–74.9)	46.7 (40.5–53.0)
Fukui	43.2	45.5	41.4	47.5	47.9	45.9	50.7 (43.5–57.8)	49.3 (41.5–56.8)	49.9 (39.7–60.1)
Yamanashi	45.8	49.4	43.4	55.4	57.1	54.2	62.2 (51.5–72.0)	62.9 (53.0–72.0)	60.5 (49.3–70.4)
Nagano	44.3	46.5	42.1	52.3	55.1	49.5	56.9 (49.5–64.4)	59.2 (48.3–69.2)	54.4 (45.3–63.1)
Gifu	37.2	41.3	32.8	48.3	52.3	44.8	57.1 (47.3–66.7)	60.8 (50.7–70.4)	54.2 (44.4–63.2)
Shizuoka	40.2	43.1	37.4	48.3	51.5	45.2	53.2 (45.5–61.1)	56.4 (48.7–63.7)	49.8 (39.8–60.1)
Aichi	37.8	41.4	34.3	46.3	49.8	43.1	52.7 (44.5–60.4)	55.6 (47.4–63.2)	50.3 (35.4–65.1)
Mie	39.3	41.6	36.8	45.7	48.6	42.4	49.7 (41.6–57.8)	53.5 (46.9–59.6)	45.3 (32.5–58.6)
Shiga	39.4	44.2	35.2	44.8	49.5	40.7	49.2 (37.2–60.6)	55.0 (40.3–69.5)	44.5 (31.8–56.8)
Kyoto	35.0	38.6	32.2	39.6	42.8	36.3	43.9 (34.8–53.1)	47.6 (33.1–61.8)	39.6 (33.9–45.9)
Osaka	29.8	32.5	27.4	40.3	43.0	37.9	48.5 (39.6–57.3)	51.7 (40.7–62.9)	45.8 (36.7–55.3)
Hyogo	34.8	40.2	29.8	43.2	47.6	39.2	50.3 (37.7–63.4)	54.1 (41.7–66.6)	46.6 (32.2–61.1)
Nara	35.8	39.5	32.5	43.3	48.0	39.7	49.7 (39.3–59.8)	54.9 (41.4–67.2)	45.6 (34.8–56.5)
Wakayama	33.7	37.1	30.0	40.6	43.8	38.5	44.6 (35.4–54.1)	48.2 (42.6–54.0)	43.1 (28.9–58.7)
Tottori	40.5	42.3	39.8	48.6	51.9	47.3	54.3 (48.3–60.1)	58.9 (50.5–67.2)	50.6 (37.8–63.5)
Shimane	43.3	45.1	40.1	51.2	53.3	49.6	56.9 (50.1–63.4)	59.7 (50.3–68.3)	56.5 (49.6–63.4)
Okayama	41.0	43.0	38.9	49.2	50.9	47.3	55.1 (49.1–61.1)	56.7 (49.1–64.3)	53.4 (47.6–59.6)
Hiroshima	37.2	39.9	34.8	44.0	49.7	38.4	48.3 (42.0–54.6)	55.4 (45.6–64.8)	41.2 (35.7–47.0)
Yamaguchi	32.8	37.5	28.3	38.3	43.5	33.0	41.6 (34.7–49.4)	47.6 (42.0–53.2)	35.8 (28.6–43.3)
Tokushima	33.5	35.6	31.4	40.5	44.2	37.3	45.3 (34.0–56.9)	50.3 (36.1–64.5)	40.9 (33.6–48.3)
Kagawa	39.6	43.1	36.6	47.9	51.1	44.6	54.0 (37.5–70.4)	57.0 (40.5–72.8)	50.7 (32.8–68.3)
Ehime	35.8	39.2	32.5	45.1	49.6	40.8	51.7 (45.8–57.4)	57.0 (50.7–63.2)	46.6 (39.4–54.0)
Kochi	35.5	37.5	33.8	48.1	50.0	46.2	56.9 (46.7–66.7)	58.5 (50.3–66.7)	55.2 (43.2–67.8)
Fukuoka	32.1	35.5	29.0	42.1	45.5	38.8	48.9 (41.4–56.3)	52.5 (41.9–63.6)	45.4 (38.5–52.4)
Saga	37.8	42.0	34.7	46.8	51.3	43.0	52.2 (39.8–63.6)	55.2 (34.7–75.0)	50.1 (37.5–63.1)
Nagasaki	32.3	36.4	28.6	39.5	41.8	36.8	44.4 (38.7–50.0)	46.0 (39.5–52.5)	42.9 (37.7–48.5)
Kumamoto	40.7	43.0	38.6	47.6	51.3	45.1	53.3 (15.8–89.4)	57.8 (13.8–92.5)	50.1 (26.5–74.0)
Oita	35.9	40.7	31.5	45.0	48.5	43.0	52.3 (43.9–60.7)	55.1 (43.3–66.7)	51.6 (43.8–59.4)
Miyazaki	34.5	38.8	31.2	44.2	49.0	40.4	50.9 (42.9–59.1)	56.1 (50.3–62.0)	46.4 (36.9–55.9)
Kagoshima	36.3	38.9	33.8	44.0	47.0	40.9	50.0 (38.2–62.0)	53.6 (39.4–67.2)	46.3 (34.5–57.4)
Okinawa	33.1	35.2	31.5	38.4	40.0	37.0	42.5 (35.8–49.1)	43.6 (38.0–49.0)	41.8 (32.5–51.3)

Adults aged 40–69 years. CrI = credible interval

Baseline (2013), current (2022), and projected (2028) coverage percentages for the total population, men, and women in each prefecture; nationwide estimates appear in bold. Projections derived from Bayesian logit regression models with 95% credible intervals (CrI). Data from Ministry of Health Comprehensive Survey of Living Conditions via e-Stat Japan. Coverage rates for adults aged 40–69 years

By 2022, nationwide coverage had risen to 45.9% (men 49.1%, women 42.8%), a median gain of 8 percentage points since 2013 (Table 1). Kochi in southern Shikoku (+ 12.6 pp), Aomori (+ 12.3 pp), and Gifu (+ 11.1 pp) grew fastest, while Tochigi (+ 4.1 pp), Fukui (+ 4.3 pp), and Kyoto (+ 4.6 pp) grew slowest. Yamagata remained the only prefecture above 60%, at 64.7%. Five prefectures remained below 40%, spanning the geographic extremes of the country from Hokkaido (38.1%) and Yamaguchi (38.3%) in the north and west to Okinawa (38.4%) in the far south, along with Nagasaki (39.5%) and Kyoto (39.6%).

**Fig. 1:**
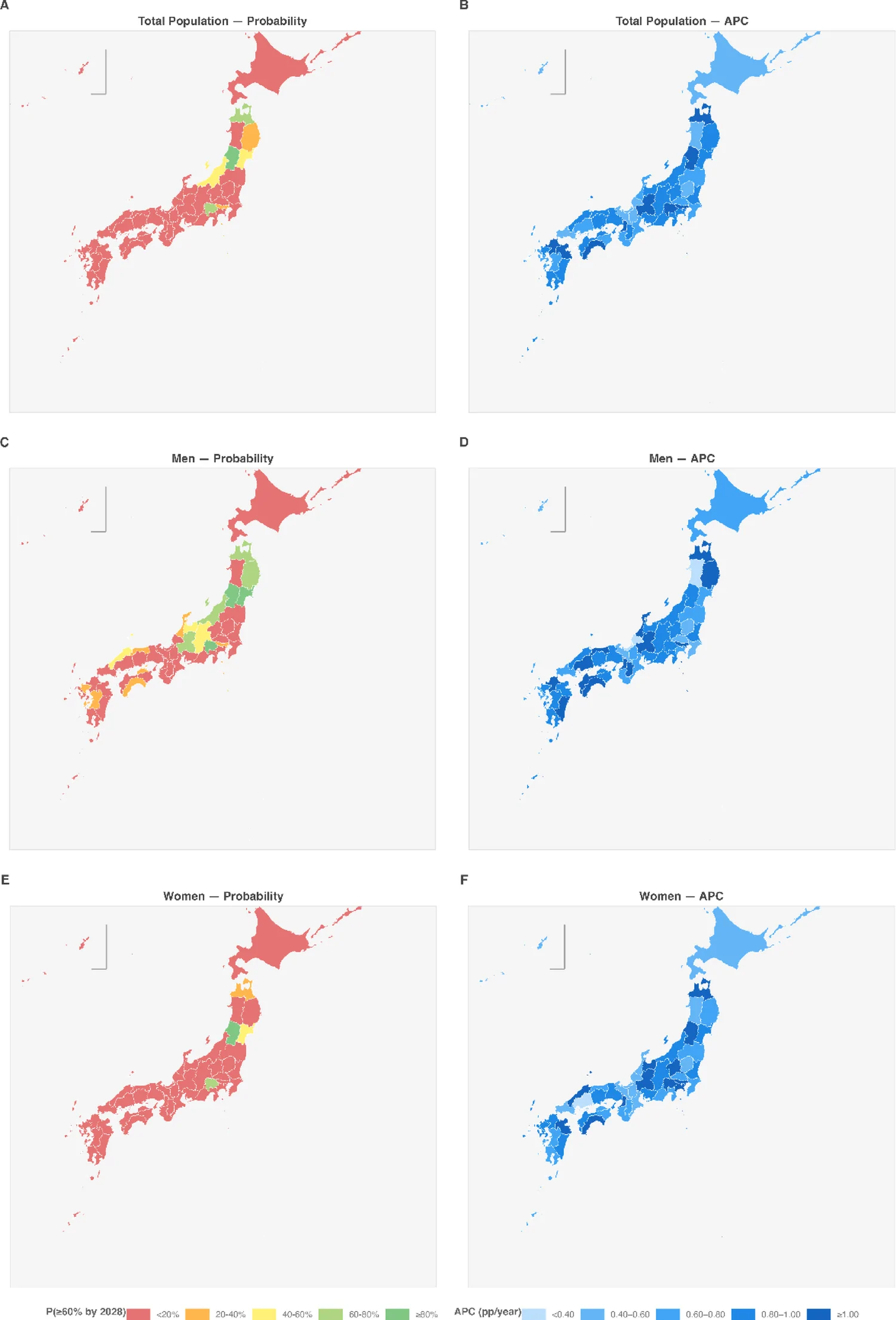
Geographic variation in CRC screening performance. Six-panel maps showing, for the total population, men, and women, the posterior probability of reaching 60% by 2028 (**A**–**C**) and the mean APC over 2013–2022 (**D**–**F**)

Nationwide APC was 0.86 percentage points per year (95% CrI 0.28–1.44) (Table 2). Prefectural APCs ranged from 0.50 in Akita (CrI − 0.09 to 1.11) to 1.37 in Aomori (CrI − 0.30 to 2.81) (Fig. 1D–F). Nine prefectures exceeded 1.0 pp/year, scattered across regions: Aomori (1.37) and Yamagata (1.09) in Tohoku, Gifu (1.25) and Tokyo (1.14) in central Japan, Kochi (1.30) in Shikoku, and Osaka (1.08) in Kinki, and Yamanashi (1.04), Fukuoka (1.01), and Oita (1.01) in Chubu and Kyushu. Akita had the slowest growth at 0.50 pp/year, with Tochigi and Fukui close behind at 0.51 pp/year. Credible intervals crossed zero in many prefectures, reflecting the uncertainty inherent in fitting trends to four observations.

**Table 2:** Projected trends and target achievement by prefecture

Prefecture	Total		Men		Women	
	APC	P(60%)	APC	P(60%)	APC	P(60%)
Nationwide	0.86 (0.28–1.44)	0.017	0.86 (0.19–1.52)	0.052	0.88 (0.22–1.50)	0.013
Hokkaido	0.54 (− 0.40–1.38)	0.010	0.62 (− 0.31–1.50)	0.023	0.46 (− 0.41–1.22)	0.006
Aomori	1.37 (− 0.30–2.81)	0.628	1.30 (− 0.55–3.01)	0.733	1.35 (0.19–2.37)	0.358
Iwate	0.89 (− 0.08–1.86)	0.287	1.15 (− 0.34–2.51)	0.752	0.72 (− 0.64–2.01)	0.129
Miyagi	0.82 (− 0.00–1.61)	0.591	0.66 (0.12–1.20)	0.809	0.94 (− 0.21–2.08)	0.420
Akita	0.50 (− 0.09–1.11)	0.026	0.38 (− 0.31–1.07)	0.044	0.44 (− 0.10–0.97)	0.010
Yamagata	1.09 (0.39–1.80)	0.988	0.87 (0.00–1.76)	0.980	1.10 (0.16–2.10)	0.972
Fukushima	0.73 (0.19–1.26)	0.032	0.75 (0.11–1.36)	0.121	0.69 (0.20–1.18)	0.013
Ibaraki	0.87 (− 0.24–1.93)	0.059	0.87 (0.17–1.51)	0.045	0.87 (− 0.72–2.28)	0.074
Tochigi	0.51 (− 0.60–1.63)	0.044	0.47 (− 0.47–1.33)	0.040	0.51 (− 1.12–2.01)	0.052
Gunma	0.92 (− 0.06–1.80)	0.058	0.80 (− 0.39–1.92)	0.122	1.02 (0.03–1.90)	0.042
Saitama	0.59 (− 0.57–1.65)	0.030	0.55 (− 0.94–1.95)	0.061	0.66 (− 0.14–1.42)	0.013
Chiba	0.62 (− 0.33–1.54)	0.033	0.53 (− 0.11–1.14)	0.021	0.72 (− 0.59–1.93)	0.050
Tokyo	1.14 (0.10–2.11)	0.263	1.01 (− 0.15–2.08)	0.390	1.27 (0.30–2.16)	0.193
Kanagawa	0.89 (0.25–1.51)	0.026	0.72 (0.10–1.35)	0.047	1.08 (0.44–1.69)	0.021
Niigata	0.92 (− 0.02–1.89)	0.467	0.94 (− 0.43–2.21)	0.777	0.88 (− 0.02–1.70)	0.128
Toyama	0.97 (0.39–1.54)	0.131	0.91 (− 0.10–1.88)	0.406	1.07 (0.18–1.96)	0.138
Ishikawa	0.77 (− 0.41–1.88)	0.059	1.08 (− 0.46–2.55)	0.382	0.58 (0.06–1.10)	0.006
Fukui	0.51 (− 0.13–1.13)	0.016	0.28 (− 0.41–0.96)	0.012	0.57 (− 0.34–1.44)	0.025
Yamanashi	1.04 (0.04–1.99)	0.780	0.88 (− 0.06–1.81)	0.850	1.09 (0.08–2.01)	0.598
Nagano	0.86 (0.19–1.53)	0.111	0.86 (− 0.16–1.81)	0.412	0.85 (0.04–1.60)	0.058
Gifu	1.25 (0.42–2.04)	0.169	1.26 (0.37–2.14)	0.625	1.30 (0.53–1.96)	0.058
Shizuoka	0.83 (0.16–1.50)	0.033	0.88 (0.21–1.53)	0.083	0.75 (− 0.12–1.56)	0.026
Aichi	0.92 (0.20–1.55)	0.028	0.95 (0.21–1.60)	0.070	0.90 (− 0.38–2.02)	0.054
Mie	0.64 (− 0.06–1.31)	0.016	0.76 (0.17–1.30)	0.022	0.49 (− 0.68–1.53)	0.020
Shiga	0.55 (− 0.54–1.51)	0.028	0.65 (− 0.67–1.96)	0.145	0.50 (− 0.65–1.46)	0.017
Kyoto	0.53 (− 0.24–1.27)	0.009	0.53 (− 0.80–1.70)	0.033	0.46 (− 0.02–0.94)	0.003
Osaka	1.08 (0.42–1.68)	0.014	1.14 (0.27–1.95)	0.042	1.04 (0.39–1.65)	0.011
Hyogo	0.89 (− 0.14–1.89)	0.042	0.85 (− 0.24–1.90)	0.090	0.92 (− 0.25–1.92)	0.029
Nara	0.84 (− 0.04–1.63)	0.024	0.99 (− 0.18–2.03)	0.112	0.75 (− 0.13–1.56)	0.015
Wakayama	0.66 (− 0.10–1.38)	0.011	0.71 (0.26–1.18)	0.005	0.74 (− 0.44–1.78)	0.022
Tottori	0.89 (0.37–1.38)	0.026	1.05 (0.30–1.81)	0.319	0.71 (− 0.42–1.77)	0.044
Shimane	0.88 (0.27–1.46)	0.091	0.94 (0.06–1.75)	0.472	1.05 (0.44–1.64)	0.084
Okayama	0.93 (0.40–1.45)	0.036	0.91 (0.22–1.58)	0.106	0.94 (0.44–1.45)	0.022
Hiroshima	0.73 (0.20–1.23)	0.008	1.05 (0.20–1.84)	0.090	0.39 (− 0.08–0.86)	0.003
Yamaguchi	0.57 (− 0.03–1.15)	0.005	0.66 (0.19–1.11)	0.005	0.47 (− 0.11–1.01)	0.003
Tokushima	0.78 (− 0.15–1.61)	0.015	0.97 (− 0.19–2.04)	0.051	0.61 (0.02–1.15)	0.004
Kagawa	0.82 (− 0.65–2.20)	0.131	0.81 (− 0.69–2.27)	0.264	0.77 (− 0.80–2.15)	0.077
Ehime	0.99 (0.51–1.44)	0.012	1.14 (0.60–1.67)	0.094	0.84 (0.27–1.39)	0.007
Kochi	1.30 (0.45–2.10)	0.170	1.30 (0.60–2.01)	0.256	1.25 (0.26–2.20)	0.126
Fukuoka	1.01 (0.42–1.54)	0.010	1.01 (0.11–1.88)	0.050	0.97 (0.45–1.44)	0.006
Saga	0.97 (− 0.09–1.88)	0.054	0.96 (− 0.82–2.63)	0.218	0.97 (− 0.08–1.93)	0.042
Nagasaki	0.75 (0.30–1.17)	0.004	0.61 (0.05–1.14)	0.005	0.86 (0.47–1.24)	0.002
Kumamoto	0.73 (− 2.34–3.07)	0.162	0.90 (− 2.38–3.53)	0.392	0.69 (− 1.33–2.30)	0.066
Oita	1.01 (0.31–1.69)	0.031	0.94 (− 0.11–1.91)	0.111	1.19 (0.61–1.75)	0.021
Miyazaki	0.99 (0.33–1.63)	0.020	1.10 (0.59–1.60)	0.053	0.89 (0.14–1.56)	0.013
Kagoshima	0.79 (− 0.21–1.74)	0.035	0.86 (− 0.39–2.03)	0.096	0.70 (− 0.32–1.55)	0.016
Okinawa	0.56 (0.00–1.08)	0.004	0.53 (0.07–0.97)	0.002	0.58 (− 0.18–1.29)	0.009

APC = annual percentage-point change (pp/yr) with 95% CrI. P(60%) = posterior probability of reaching 60% coverage by 2028

Bayesian logit regression, prefecture-level data, 2013–2022

For each prefecture and sex: mean APC with 95% credible interval and posterior probability of reaching 60% coverage by 2028. APC = annual percentage-point change in coverage (percentage points per year). Projections derived from Bayesian logit regression models using prefecture-level surveillance data (2013–2022). Nationwide estimates calculated using population-weighted posterior sample aggregation

Projected mean nationwide coverage in 2028 was 51.9% (CrI 44.9–58.8%), falling 8.1 percentage points short of the target (Fig. 1A–C, Table 2). Projected coverage was highest in the northeast (Tohoku mean 59.8%) and central Japan (Chubu 55.8%), and lowest in the Kinki region around Osaka and Kyoto (48.0%) and in Hokkaido (42.4%). The nationwide probability of reaching 60% by 2028 was 0.017 (men 0.052, women 0.013). Only Yamagata exceeded the 80% probability threshold for the total population (P = 0.988). For men, Yamagata (0.980), Yamanashi (0.850), and Miyagi (0.809) also exceeded this threshold. For women, only Yamagata (0.972) did. Forty of 47 prefectures (85%) had probabilities below 0.20, and 29 (62%) fell below 0.05.

**Fig. 2:**
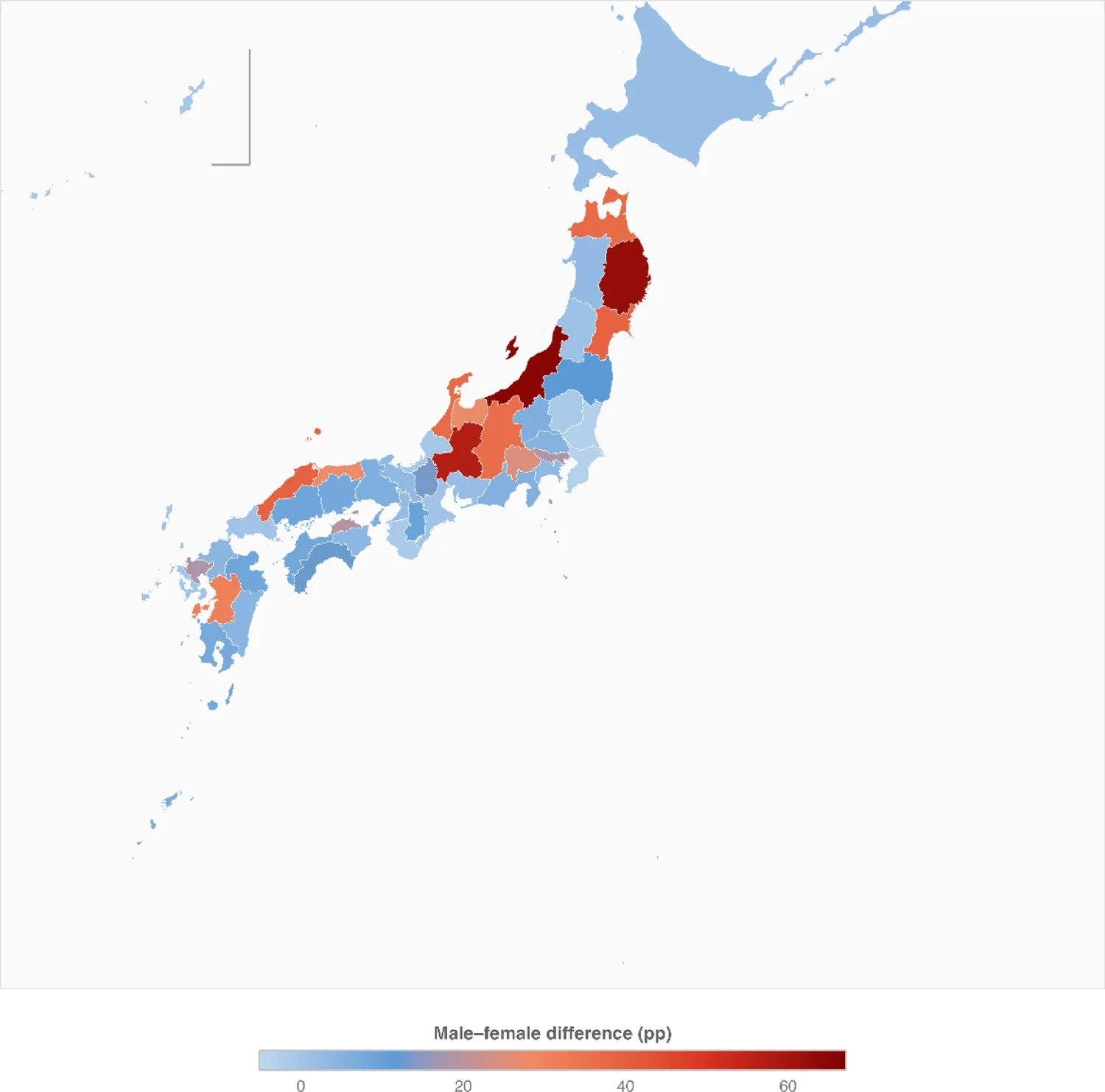
Gender Disparities in Target Achievement Probability by Prefecture. Choropleth map showing the absolute difference in 2028 target achievement probability (probability for men minus probability for women) for each prefecture. Darker shading indicates larger sex disparities, with men having higher target achievement probabilities than women in 41 of 47 prefectures

Men had higher target achievement probabilities than women in 41 of 47 prefectures, with a median gap of 8.0 percentage points (Fig. 2, Table 2). The largest gaps clustered along the Japan Sea coast and central Honshu: Niigata (64.9 pp), Iwate (62.3 pp), and Gifu (56.7 pp). In Gifu, men had a probability of 0.625 compared with 0.058 for women. Women lagged men by a median of 6 years in projected achievement. Three prefectures exceeded the 80% threshold for men versus one for women. Six prefectures (Ibaraki, Tochigi, Chiba, Fukui, Wakayama, Okinawa) showed slightly higher probabilities for women, though all remained below 0.08.

Out-of-sample validation (training on 2013–2019, predicting 2022) gave a median MAE of 2.05 percentage points (mean 2.14 pp), with 100% credible interval coverage across all 141 prefecture-sex combinations (eFigure 1, eTable 1). The best predictions were near-exact, such as Iwate total (MAE 0.003 pp) and Yamagata total (0.02 pp). The largest errors occurred in Aomori men (7.0 pp overprediction) and Saga men (6.7 pp underprediction). Nearly three quarters of predictions had MAE below 3 pp (eFigure 2). As a sensitivity analysis, we compared results from the restricted dataset (2013–2022) with those obtained using all six survey waves (2007–2022). Including the earlier waves, which captured a 12-percentage-point jump in coverage between 2007 and 2013, produced systematically higher target achievement probabilities across all prefectures and higher out-of-sample prediction error (eFigure 3).

## Discussion

This study examined trends in CRC screening and projected Japan's progress toward the government's target of 60% coverage by 2028. Nationwide coverage rose from 37.9% in 2013 to 45.9% in 2022. Within these overall trends, substantial regional and gender disparities were evident. Only Yamagata has exceeded 60%. Three prefectures exceeded the 80% probability threshold for men by 2028 (Yamagata, Yamanashi, Miyagi), but only Yamagata did for women. These findings suggest the need for prefecture-specific and gender-sensitive interventions to achieve and potentially exceed the Health Japan 21 target for CRC screening.

In the latest survey year, CRC screening uptake was 45.9%, consistent with earlier reports of suboptimal participation in Japan [7]. In global comparison, Japan ranks among the lowest of developed countries in CRC screening coverage [19]. Participation in cancer screening in Japan remains low, with coverage for all major cancers falling short of the 60% target. Previous evaluations have suggested that one contributing factor is the fragmented nature of the delivery system [20, 21]. Screening is provided through municipalities, workplaces, and private services; however, each operates independently, leaving no unified mechanism to track or ensure participation. As a result, national estimates rely largely on self-reported surveys [20]. Workplace programs reach many people, but participation depends on individual employer policies. Meanwhile, cost remains a barrier at the municipal level, with only a minority of municipalities fully subsidizing screening. The central government could consider adopting a unified national management policy to improve equity, accountability, and data integration, addressing inconsistencies in access and financing.

In addition to these general challenges in cancer screening, several factors unique to CRC screening further limit participation. In Japan, between 2007 and 2022, CRC screening showed the largest increase in participation among major cancer screenings, yet coverage still remains lower than that of lung cancer [22]. Social determinants of low participation include younger age (< 60 years), female sex, lower education and income, and unmarried or unemployed status [7]. Inequalities in CRC screening are greater than in other cancer screenings [23]. At the individual level, barriers vary by screening modality but can be grouped into informational (limited awareness and doubts about test efficacy), attitudinal (fatalistic views of cancer, fear of diagnosis and treatment, embarrassment), and practical (low motivation, competing demands, scheduling difficulties) [24, 25]. Cost also plays a role; in a 2023 Cabinet Office survey, 23% of respondents cited financial burden as a reason for not undergoing cancer screening [26]. Although qualitative studies specific to Japan are scarce, global barriers are likely relevant and may be accentuated by local factors. For example, evidence from Asian American populations suggests that fear of cancer and fatalistic attitudes deter participation, and similar cultural perceptions may influence Japanese populations [27]. Practical barriers also include the burden of specimen preparation, such as stool collection for FIT, which may be complicated by Japan's high prevalence of constipation among adults over 40 [28]. Additionally, bowel preparation for colonoscopy is often met with reluctance due to its invasiveness and discomfort, further limiting follow-up after a positive FIT [29]. Broader efforts are also required to strengthen awareness and evidence from qualitative research to identify barriers specific to the Japanese population.

Regional disparities in CRC screening and outcomes are well recognized. Data from the MHLW CSLC consistently show prefectural variation in screening coverage, confirming descriptive differences across regions [8]. Both incidence and mortality also vary substantially across prefectures, with Aomori standing out as the prefecture with the highest burden [30]. Between 1990 and 2021, reductions in CRC mortality contributed minimally to gains in life expectancy in Japan, with prefectural differences ranging from zero to only 0.2 years, underscoring persistent inequities [9]. Similar patterns are observed globally; for example, in the United States, both CRC screening participation and outcomes vary substantially by state [31].

Several structural and policy factors contribute to the wide prefectural disparities in CRC screening. Cancer control in Japan is decentralized: the central government sets broad direction through the Basic Plan to Promote Cancer Control Programs, prefectures prepare their own plans, and municipalities serve as frontline providers [26]. Financial arrangements may also contribute to this variation. Although CRC screening fees are relatively low, financial concerns remain a reported barrier (as noted above), reflecting differences across municipalities. Only about 10% fully subsidize screening, around 70% offer partial subsidies, and the remainder require full payment [32]. Governance capacity also varies, with only 17 prefectures reporting municipal-level monitoring of cancer control and just 10 integrating cancer control into broader medical [33]. Miyagi Prefecture, known as the birthplace of group cancer screening, showed a higher chance of reaching the target in our analysis. It has set a screening rate goal of over 70% above the national benchmark and supports this through national and prefectural subsidies, as well as systems for residents without access to workplace screening [34]. Such initiatives may help explain Miyagi's relatively successful efforts in cancer screening. While most prefectures follow national guidelines, each should assess local needs to adapt policies accordingly, and draw lessons from high-performing regions to accelerate progress toward the Health Japan 21 target.

Gender differences are an important dimension of CRC screening. Women have lower adenoma prevalence, more right-sided cancers, and reduced fecal hemoglobin levels, which makes FIT less sensitive in detecting lesions; nevertheless, evaluations show that FIT screening is equally cost-effective for both men and women [35, 36]. Our analysis found that women in Japan had consistently lower participation rates than men at both national and prefectural levels. This aligns with prior research in Japan [7, 22, 23]. In contrast, global evidence on gender differences in FIT-based CRC screening is mixed: a major meta-analysis and a UK study reported lower uptake among men [25, 37], whereas studies from the United States show inconsistent results, with some finding lower participation among women and others no substantial difference [38, 39]. These variations may reflect systemic and cultural factors, as well as differences in study populations, since most international research has focused on adults aged 50 and older, while in Japan screening is recommended from age 40.

Explanations for lower female participation internationally emphasize psychosocial and systemic factors, including the misperception of CRC as a 'man's disease,' heightened embarrassment and fear of colonoscopy following a positive FIT, and a lack of strong physician recommendations [40]. In Japan, lower female uptake appears more consistent, likely reflecting a combination of structural and cultural barriers. Most screenings are delivered through the workplace, which is associated with higher participation among workers than non-workers [41]. Workplace screening primarily reaches regular employees, and the majority of employed women in Japan work in non-regular positions [42]. Employment type and company size are directly associated with screening participation, with non-regular workers in smaller companies showing significantly lower uptake [43]. Women outside the regular workforce, including homemakers, must rely on municipal screening, where resource availability and outreach intensity vary across prefectures [14]. The observed sex disparities in projected target achievement may therefore reflect structural differences in access to screening rather than differences in health-seeking behaviour. The variation in female participation across prefectures suggests that regional socioeconomic conditions, lifestyle risk factors, and the effectiveness of local outreach may also shape the gender gap. To advance the Health Japan 21 goal, policies must go beyond system-level supports and adopt gender-sensitive initiatives, including outreach tailored to homemakers and non-regular workers, awareness programs designed specifically for women, and communication strategies that directly address women's concerns and cultural perceptions.

This analysis used self-reported screening participation from the triennial CSLC. Self-reported data may be affected by recall bias, social desirability bias, and misclassification between screening and diagnostic examinations. The measure also likely captures screening across multiple channels, including municipal, workplace-based, and opportunistic services, rather than organized screening alone. These issues may affect the absolute level of estimated uptake. However, because the CSLC is a standardized and consistently collected national data source, it remains useful for comparing temporal trends and regional and sex disparities. In addition, the triennial survey schedule yields sparse observations, requiring the model to bridge gaps between waves; this may flatten or exaggerate estimated changes if underlying trends were not smooth. Furthermore, we relied on published prefecture–sex point estimates rather than underlying counts or microdata. Consequently, we could not model binomial variation or weight observations by sample size, and instead used logit-transformed rates with a Gaussian error structure. This may not fully capture heteroscedastic uncertainty when sample sizes vary, potentially affecting precision. We also fitted models independently for each prefecture–sex stratum rather than using a hierarchical framework, which may reduce efficiency and increase sensitivity to noise. A hierarchical model with partial pooling would likely yield more stable forecasts and target attainment estimates. The present analysis did not include prefecture-level covariates such as demographic composition, socioeconomic factors, or healthcare infrastructure, as the primary aim was to evaluate the probability of meeting a specific policy target from observed trends. Future research could incorporate such variables within a multivariate framework to better understand drivers of variation in screening uptake. The model assumes a linear trend on the logit scale, which may not capture potential non-linear changes in screening uptake over time due to factors such as population shifts, policy changes, or saturation effects [44]. Accordingly, projections should be interpreted as extensions of recent trends rather than precise forecasts. However, this parsimonious specification avoids overfitting given the limited data and provides a transparent baseline projection to inform and evaluate future policy changes. The CSLC questionnaire underwent documented layout changes around the 2010–2013 period, which may have contributed to the observed structural break in coverage estimates. Restricting the analysis to 2013–2022 data mitigates but does not eliminate the possibility that measurement artifacts affect observed trends.

CRC screening coverage in Japan rose from 37.9 to 45.9% between 2013 and 2022, but the 60% target will not be reached nationally until 2042 if current trends hold. Only Yamagata has exceeded 60%, and most prefectures have less than a 5% chance of doing so by 2028. Women lag men by a median of 6 years in projected achievement. Stronger national coordination, support for underperforming prefectures, and gender-sensitive strategies will be needed to close the remaining gap across all populations.

## Supplementary Information

Below is the link to the electronic supplementary material.

Supplementary Material 1.

Supplementary Material 2.

Supplementary Material 3.

Supplementary Material 4.

## Data Availability

The data used in this study are publicly available on e-Stat Portal Site of Official Statistics of Japan (https://www.e-stat.go.jp/).
