# Supplementary material for: Forecasting colorectal cancer screening in Japan: regional and sex disparities threaten national targets
Source: Int J Clin Oncol. 2026 Jul 27;31(10):2415–25. doi: 10.1007/s10147-026-03150-8 (PMC13615035; doi:10.1007/s10147-026-03150-8)
Supplement: Supplementary file 1 [file 10147_2026_3150_MOESM1_ESM.docx]

Table 1: eTable 1. Out-of-sample model validation: training 2013–2019, validation 2022

| Group | n | MAE | ME | RMSE | MAPE | Coverage |
| --- | --- | --- | --- | --- | --- | --- |
| Men | 46 | 2.39 | 1.52 | 2.39 | 4.8 | 100.0% |
| Total Population | 47 | 2.00 | 1.50 | 2.00 | 4.4 | 100.0% |
| Women | 47 | 2.08 | 1.37 | 2.08 | 4.8 | 100.0% |
| **All** | **140** | **2.16** | **1.46** | **2.16** | **4.7** | **100.0%** |
| MAE = mean absolute error (pp). ME = mean error (pp; positive = overestimation). RMSE = root mean square error (pp). MAPE = mean absolute percentage error. Coverage = proportion of observed values within 95% credible intervals. | | | | | | |
| Models trained on 2013, 2016, 2019 data, validated against observed 2022 values. | | | | | | |
